# Attitudes toward Sustainable Development Goals (SDGs) and civic engagement among Spanish university students: the mediational role of flourishing

**DOI:** 10.3389/fpsyg.2026.1859404

**Published:** 2026-07-30

**Authors:** Carmen Pozo Muñoz, Blanca Bretones Nieto, Yesid José Ortega Pacheco, Yeison David Gallo-Barrera

**Affiliations:** 1 University of Almeria, Almería, Spain; 2 Aix Marseille Univ, LPS, ISSPAM, Aix-en-Provence, France; 3 Universidad Cooperativa de Colombia, Cartago, Colombia

**Keywords:** civic engagement, cross-sectional study, flourishing, SDGs, university students

## Abstract

The objective of the present study was to examine the mediating effect of flourishing on the relationship between positive attitudes toward the Sustainable Development Goals (SDGs) and civic engagement among university students from Andalusia, Spain. A total of 215 students participated (51 men, 164 women). SPSS PROCESS Macro (Model 4) with bootstrapping (95% CI) was used to determine the mediation effect between variables. Additionally, Spearman's correlations, common method bias, and internal consistency of the scales were assessed; Harman's single-factor test confirmed that common method bias was not a threat (accounting for only 22.43% of the variance), and all measurement scales exhibited robust internal consistency (alpha ≥ 0.76). Results indicated that attitudes toward SDGs significantly predict civic engagement (β = 0.315, *p* < 0.01), maintaining a direct effect after including flourishing (β = 0.212, *p* < 0.01). Flourishing mediates this relationship (indirect effect = 0.103), accounting for 32.7% of the total effect. It is concluded that pro-SDG attitudes foster civic engagement both directly and through flourishing. Interventions promoting psychological wellbeing can enhance sustainability-oriented civic participation among university students.

## Introduction

Civic engagement (CE) is operationally defined as the set of individual and collective actions aimed at identifying, addressing, and resolving issues of public concern (Delli Carpini, 2000). This definition positions CE as a proactive behavior, essential for the health of the public sphere, representing, in essence, the active participation of individuals oriented toward the common good of society (Ehrlich, 2000). The conceptual articulation of CE is intrinsically relational, encompassing structural elements such as trust in social institutions and individuals, adherence to norms of reciprocity, and integration into active civic networks (Delli Carpini, 2000). Participation in these social processes is crucial, as it facilitates individuals acquiring the skills and knowledge necessary to generate meaningful and sustainable change within their local environment (Bobek et al., 2009; Fenn et al., 2023).

The manifestations of civic engagement are diverse, covering a broad spectrum ranging from individual acts of volunteerism (civic engagement) to participation in electoral processes (electoral engagement), political activism (protest), or new online engagement modalities through digital platforms (Fenn et al., 2024). While a universal definition remains elusive, CE generally involves efforts to assist others, address specific problems, or collaborate on resolving shared challenges, which entails reciprocal networks, active participation, and a developed sense of social responsibility (Fenn et al., 2023). Perhaps the definition proposed by Adler and Goggin (2005) remains one of the clearest, characterizing engagement as the way in which citizens participate in the life of a community to improve conditions for others or to help shape the community's future.

Emerging adulthood (EA), a period spanning approximately 18–29 years (Arnett, 2007), represents a critical stage in the life cycle for the formation and consolidation of personal, political, and civic identities (Fenn et al., 2024; Lerner et al., 2014). During this transitional phase, young people solidify the values and participation patterns that will define their adult trajectory. The university student population is of relevance to the study of civic engagement. Higher education institutions traditionally serve as key socialization environments, where young people act as agents of social change (Wakkee et al., 2019). This academic context is conducive to the development of social competencies, the improvement of life satisfaction, and the fostering of interpersonal trust and trust in authorities and institutions (Hansen and Lehmann, 2006; Morawska-Jancelewicz, 2022).

Civic engagement manifested during adolescence is associated with a greater likelihood of future participation in civic activities and is directly linked to better academic, socioemotional, civic, and behavioral outcomes in emerging adulthood (Chan et al., 2014). Longitudinal research supports the conclusion that early CE is associated with more positive health profiles later in life. Activism, volunteering, and voting in young adulthood are consistently related to better mental health, higher educational attainment, and superior personal and family income levels (Ballard et al., 2019).

In recent years, there has been an intensification of research focused specifically on the relationship between CE, health, and wellbeing in the population of university students and young adults (Albright et al., 2020; Fenn et al., 2021; Ng et al., 2025). Active participation in civic life strengthens the individual's sense of connection with society and their role within it. Furthermore, strong social networks, resulting from civic engagement, have been found to be associated with better physical health and superior general wellbeing (Ding et al., 2015). Engagement in civic activities, such as volunteering, has been correlated with favorable mental health outcomes, including a reduction in depressive symptoms and a decrease in participation in risky health behaviors, as well as an increase in psychological wellbeing, especially among young people (Ballard et al., 2019).

### Civic engagement and sustainable development goals

In the present study, we have focused on civic engagement around a topic of particular interest now, the Sustainable Development Goals (SDGs) incorporated in the United Nations' 2030 Agenda. Universities emerge as an Agent of Change (AOC) in its immediate community through mutually beneficial relationships within its local context. Moreover, with the transfer of knowledge between Higher Education Institutions (HEIs) becoming increasingly consistent, there is significant potential to expand the impact of these collaborations globally and across a wide variety of local contexts (O'Dwyer et al., 2023).

The 2030 Agenda for Sustainable Development was adopted in 2015 by all member states of the United Nations (UN) as a plan of action to achieve a more sustainable world in the context of combating climate change. Composed of 17 Sustainable Development Goals (SDGs) and 169 targets, this Agenda represents the continuation of the first international initiative to address global problems, the Millennium Development Goals (2000–2015). The 17 SDGs developed in the 2030 Agenda by the UN propose a path toward a more equitable society. Universities, through their role in human development, knowledge production, and innovation, are key spaces for creating awareness within the university community about the need to achieve these goals (United Nations General Assembly, 2015).

Universities are crucial platforms for raising awareness and sensitizing society about the need to advance toward sustainability, possessing significant potential to contribute to the fulfillment of the Sustainable Development Goals (SDGs) in all their spheres of action. Universities, through their educational, research, transfer, and management functions, are at the forefront of generating perspectives that contribute to the development of a more sustainable culture (Carlsen and Bruggemann, 2022).

Furthermore, HEIs that prioritize student wellbeing have the potential to indirectly foster civic engagement. Campuses with a strong emphasis on wellbeing often exhibit greater student participation in community and service-oriented activities. The university is thus conceived as a civic agent that promotes not only engagement at a general level but also the UN SDGs (Galvão et al., 2024). In this context, engagement was found to give a contribution, through active learning, in the development of a more balanced perspective on sustainability, not only or predominantly including environmental concepts, but also social and economic ones (Hong et al., 2023). Knowledge is people's perceptions of certain issues, such as the SDGs. Attitude is then what they feel about the SDGs and practice can be the result of their feelings and what they do about it (Kaliyaperumal, 2004).

Despite this commitment and interest on the part of universities, recent studies indicate a lack of awareness among university students regarding the SDGs, and a failure to integrate them into university curricula and to engage in their promotion as activities of their civic engagement (Alcántara-Rubio et al., 2022; Hong et al., 2023). To achieve these goals of global reach, it is expected that the majority of the population knows the SDGs, recognizes their importance, and finds application for such goals in any professional context, as well as in personal lifestyle. However, participation of universities is essential.

### The role of flourishing

Keyes (2005) conceptualized flourishing as a state of elevated emotional, psychological, and social wellbeing. Flourishing, distinguished by the comprehensive development of individual capabilities and satisfaction with life, exhibits a close relationship with engagement (Seligman, 2011). Individuals who demonstrate high levels of “flourishing” tend to possess richer and more satisfying social connections, experience fewer limitations in their daily activities, and enjoy better health outcomes (Catalino and Fredrickson, 2011; Huppert and So, 2013; Keyes et al., 2010). Flourishing encompasses six interrelated attributes: meaning, positive relationships, engagement, competence, positive emotion, and self-esteem (PERMA model; Seligman, 2011).

Research indicates that individuals who are flourishing are significantly more likely to engage in prosocial behaviors (Nelson and Padilla-Walker, 2013; Liu et al., 2025). Those who flourish tend to have stronger social relationships, fewer limitations in their daily activities, and better health, all of which facilitate their participation in helping others (Keyes, 2002; Catalino and Fredrickson, 2011; Huppert and So, 2013).

While some evidence suggests a direct relationship between civic engagement and flourishing (Datu, 2018), factors such as purpose in life and self-efficacy (both variables encompassed within the broader construct of flourishing) may play an important mediating role; flourishing serves as a healthy coping strategy (Nuangchalerm, 2014; Dolan, 2022). So, individuals who score high on flourishing measures have been found to have more and better social relationships, fewer limitations in their daily activities, and enjoy better health (Catalino and Fredrickson, 2011; Huppert and So, 2013; Pozo et al., 2021). Research on civic engagement has proposed that variables such as meaning in life and self-efficacy can attenuate or strengthen the relationship between engagement and well-being (Datu, 2018; Fong and To, 2022).

The discipline of positive psychology has extensively examined the benefits of meaning in life, or a connection to something that transcends the self, and that relates to civic engagement and wellbeing among young adults. Seligman (2011) defines the concept known as “flourishing,” which relates to psychological and subjective wellbeing. This concept has also been evaluated alongside the experience of emotions (positive and negative) with the goal of creating new scales to measure wellbeing (Hone et al., 2014; Tang et al., 2016). However, recent research has shown that flourishing is more than an outcome of wellbeing; it can function as a proactive way of behaving and a healthy coping strategy, enabling individuals to face difficult situations with fewer limitations and barriers (Catalino and Fredrickson, 2011; Huppert and So, 2013; Pozo and Bretones, 2019).

Flourishing has been associated with a range of positive outcomes in students' lives, including improved mental health, more satisfying interpersonal relationships, and enhanced academic performance (Keyes, 2002; Keyes et al., 2010). Within this framework, prosocial behaviors can function as catalysts for flourishing, fostering positive emotions and high-quality interpersonal connections (Fredrickson, 2004).

Although flourishing has occasionally been treated as a direct outcome of prosocial behavior or community engagement, robust theoretical and empirical foundations support its role as an active mediator that facilitates and sustains social participation. The 17 SDGs and their associated targets propose a pathway toward a more equitable and viable society; when emerging adults internalize these global goals as personal values, they develop altruistic value orientations.

Pro-SDG attitudes are theoretically positioned prior to flourishing in this model because they provide the axiological framework and ethical compass that direct the eudaimonic capital of flourishing toward generalized civic engagement. Thus, maintaining positive attitudes toward sustainability is not merely a cognitive endorsement of international policies, but a source of psychological capital that directly sustains flourishing dimensions, such as self-acceptance, optimism, positive relationships, and a sense of social contribution (Fenn et al., 2021).

By condensing optimal psychological health, Flourishing operates as an integrative mechanism (Sagiv et al., 2022); mobilizes the sense of purpose and social interconnection skills to overcome barriers to civic participation. Preliminary studies in emerging adults have already found that flourishing is positively related to social participation and civic consciousness (Zhao et al., 2025). Wellbeing interventions in U.S. colleges: a scoping review from a positive higher education perspective. *Front. Psychol*. 16:1601955. doi: 10.3389/fpsyg.2025.1601955. However, a formal test of mediation is required to isolate the pathway that demonstrates how eudaimonic health drives social engagement.

### Current study

**Figure 1:**
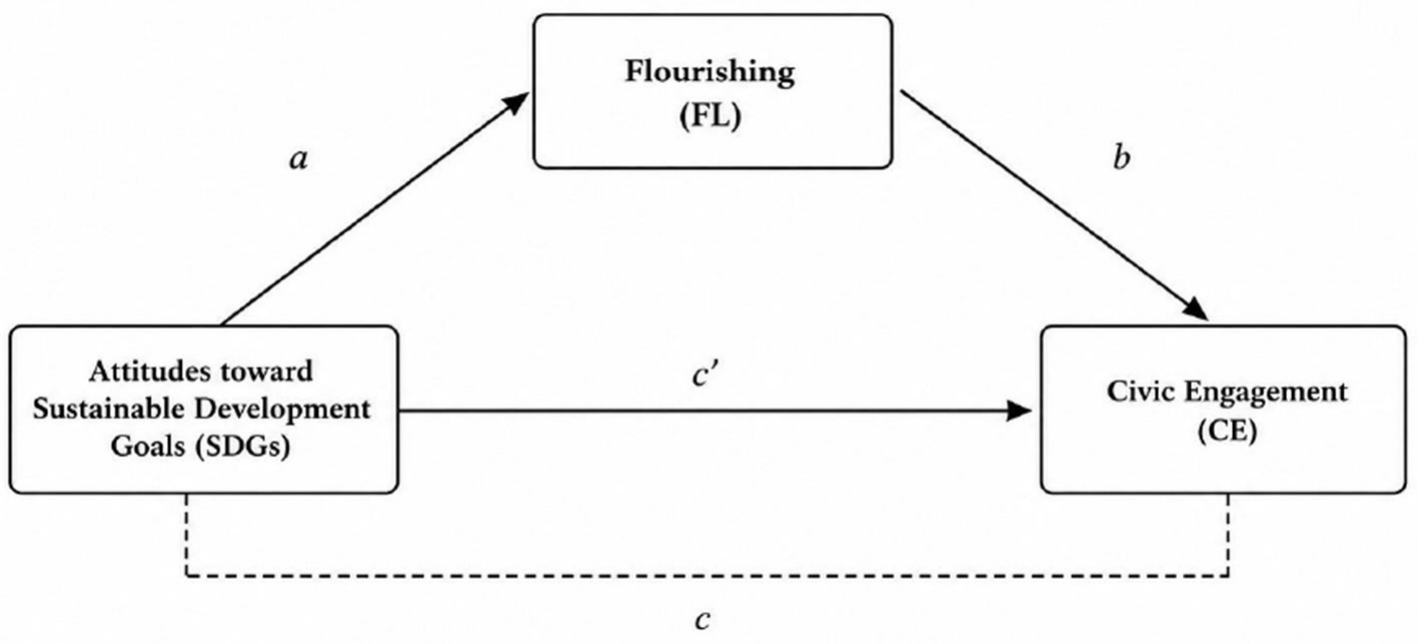
Theoretical model of the mediation of flourishing between attitudes toward the Sustainable Development Goals (SDGs) and civic engagement. a, path from attitudes toward the SDGs to flourishing; b, path from flourishing to civic engagement; c', direct effect of attitudes toward the SDGs on civic engagement controlling for flourishing; c, total effect.

The present study aims to investigate the mediating role of flourishing between attitudes toward SDGs and civic engagement among university students. We propose three hypotheses: (H1) positive attitudes toward SDGs increase civic engagement, (H2) students with more positive attitudes toward SDGs will experience higher levels of flourishing, and (H3) flourishing is a significant mediator between attitudes toward SDGs and civic engagement. Figure 1 shows the theoretical model that was tested. This research contributes to the growing body of literature on positive psychology, civic engagement, and sustainable development by examining the interplay between these constructs in a university student population. Understanding these relationships can inform interventions and educational programs aimed at fostering civic engagement and promoting wellbeing among young adults.

## Materials and methods

### Participants

All information was collected through a questionnaire administered via the LimeSurvey platform. Data were gathered from a random sample of university students from various degree programs at a public university in Andalusia, Spain. A cluster sampling approach was employed, in which each classroom was a randomly selected cluster. The inclusion criteria were: (a) being over 18 years of age, (b) having an active enrollment in an academic program, and (c) residing in a city within Andalusia. Students with cognitive difficulties that prevented the measurement were excluded.

The study was conducted in accordance with the 1975 Helsinki Declaration (as revised in WMA, 2013). All participants signed an informed consent form. Initially, the translation of the various measurement scales were conducted. After eliminating invalid questionnaires due to missing responses, a total of 215 valid questionnaires were obtained for the present study (51 men, 164 women; age *M* ± SD = 22.2 ± 4.96).

### Measures

#### Attitudes toward Sustainable Development Goals (SDGs)

The Attitudes on Sustainable Development Goals subscale from the KAP questionnaire (Afroz and Ilham, 2020) measures university students' positive perceptions and opinions toward key SDGs themes including poverty reduction, health services, environmental concerns, gender equality, and sustainability. It comprises 14 items using a five-point Likert scale (1 = Strongly Disagree to 5 = Strongly Agree), with an example item being “Environmental problems are a matter of my concern.” The scoring range is 14 to 70, with reverse-scored negative items for consistency. The internal consistency of the scale was adequate in the present study, with Cronbach's alpha of 0.83.

#### Civic engagement (CE)

The Civic Engagement subscale (Wodika and Middleton, 2020) measures students' perceptions of involvement in local and home communities, sense of empowerment to address societal issues, enjoyment of volunteering, prioritization of service-learning, and post-graduation community engagement. It consists of seven items rated on a five-point Likert scale (1 = Strongly Disagree to 5 = Strongly Agree), with an example item being “I feel involved with my local community where I attend school.” The scoring range spans 7 to 35. Cronbach's alpha in the original scale was 0.74, and in the present study it was 0.76.

#### Flourishing (FL)

Created by Diener et al. (2009) as a new tool to measure wellbeing which comprises eight items dealing with different areas, such as relationships, self-esteem, purpose, and optimism. In this study, to assess personal strengths-growth, the Spanish Version of the Flourishing Scale (Diener et al., 2009) was introduced. It contains eight items. Cronbach's alpha in the original scale is 0.87, whereas it is 0.84 in the present study. It is worth highlighting the cross-cultural research of Pozo et al. (2016), in which Colombian and Spanish university students participated, satisfactory psychometric properties (α Colombian = 0.88; α Spanish = 0.85), good levels of reliability and validity were found. Likewise, the unidimensionality of the construct and its invariance in the two samples was corroborated through a multigroup analysis. Recent studies have evidenced the adequate psychometric properties of the scale in the Spanish population (Pozo and Bretones, 2019; Pozo et al., 2021).

Regarding the translation and adaptation of the scales, *Attitudes toward the SDGs*, was adapted from the KAP questionnaire (Afroz and Ilham, 2020). A standard forward-backward translation was carry out to adapt the items into Spanish. In our sample, the scale yielded a robust internal consistency of alpha = 0.83. *Civic engagement* (CE): the subscale by Wodika and Middleton (2020) was translated into Spanish following the same forward-backward translation method. The translated scale showed satisfactory reliability in our study (alpha = 0.76), exceeding the original scale's reported alpha of 0.74. *Flourishing* (FL): we utilized the pre-existing, validated Spanish version of Diener's Flourishing Scale (Diener et al., 2009). We have added references to its psychometric validation in Spanish and Colombian university populations (e.g., Pozo et al., 2016, 2021; Pozo and Bretones, 2019), which corroborated its unidimensional structure and measurement invariance. In our current sample, the Spanish adaptation demonstrated strong reliability (alpha = 0.84).

### Statistical analysis

All statistical procedures were performed using SPSS version 26. Harman's single-factor test (Aguirre-Urreta and Hu, 2019) was employed to assess common method bias, and Spearman (2010) correlation was used to examine the relationships among the main variables before the mediation model, given their ordinal level of measurement (Likert scales) and the observed deviation from normality. This coefficient is robust to non-linear relationships and outliers and allows the verification of the direction of bivariate associations before estimating the model's direct and indirect effects.

The theoretical model shown in Figure 1 was tested using a simple mediation analysis with the SPSS PROCESS macro, with Model 4 selected for the present study (Hayes, 2012). Three paths corresponded to the study's hypotheses. First, the total effect of attitudes toward the SDGs on civic engagement (path c) tested H1. Second, the effect of attitudes toward the SDGs on flourishing (path a) tested H2. Third, the indirect effect of attitudes toward the SDGs on civic engagement through flourishing (path a × b) tested H3, the mediation hypothesis. The significance of this indirect effect was assessed using a bias-corrected bootstrap method with 5,000 resamples, with mediation supported when the resulting 95% confidence interval did not include zero. The model also estimated the effect of flourishing on civic engagement, controlling for attitudes toward the SDGs (path b) and the direct effect of attitudes toward the SDGs on civic engagement after accounting for the mediator (path c').

## Results

### Common method bias

Two methods were employed to address common method bias. First, a procedural control method was implemented, following robust protocols to ensure respondent anonymity, confidentiality, and appropriate use of data solely for research purposes. Second, Harman's single-factor test revealed that seven factors had eigenvalues greater than 1, with the first factor accounting for only 22.43% of the explained variance, a value substantially below the 40% threshold criterion (Aguirre-Urreta and Hu, 2019). Thus, common method bias did not significantly influence the relationships between variables in the present study.

### Descriptive and correlation statistics

Table 1 presents the means, standard deviations, and correlations among the study variables. Spearman's correlation analysis confirmed that positive attitudes toward the Sustainable Development Goals exhibited a weak, positive, and significant association with civic engagement and flourishing. Likewise, flourishing showed a moderate, positive, and significant correlation with civic engagement.

**Table 1:** Means, standard deviation, and correlations.

Variables	*M* ±SD	1	2	3
1. SDGs	4.30 ± 0.45	1		
2. CE	4.01 ± 0.58	0.410^**^	1	
3. FL	3.41 ± 0.62	0.296^**^	0.533^**^	1

^**^p<*0.01*.

### Results of the mediating effect of flourishing

The study utilized the SPSS PROCESS Macro (Model 4) by Hayes (2012) to determine the mediating effect of flourishing on the relationship between attitudes toward the Sustainable Development Goals (SDGs) and civic engagement (CE). The results (Table 2) indicate that the SDGs were a positive and significant predictor of CE (β = 0.315, *t* = 6.865, and *p* < 0.01), with a 95% CI of [0.225, 0.406]. Additionally, the direct predictive effect of the SDGs remained significant after including the mediator (β = 0.212, *t* = 5.160, and *p* < 0.01), with a 95% CI of [0.131, 0.293].

**Table 2:** Mediation model of flourishing.

Outcome variable	Predictor variable	Fitting index			Coefficient significance	
		*R*	*R* ^2^	*F* (df)	β	*t*
CE		0.426	0.181	47.124 (1)		
	SDGs				0.315	6.865^**^
FL		0.285	0.081	18.851 (1)		
	SDGs				0.227	6.532^**^
CE		0.633	0.400	70.789 (2)		
	FL				0.454	8.804^**^
	SDGs				0.212	5.160^**^

^**^p<*0.01*.

**Figure 2:**
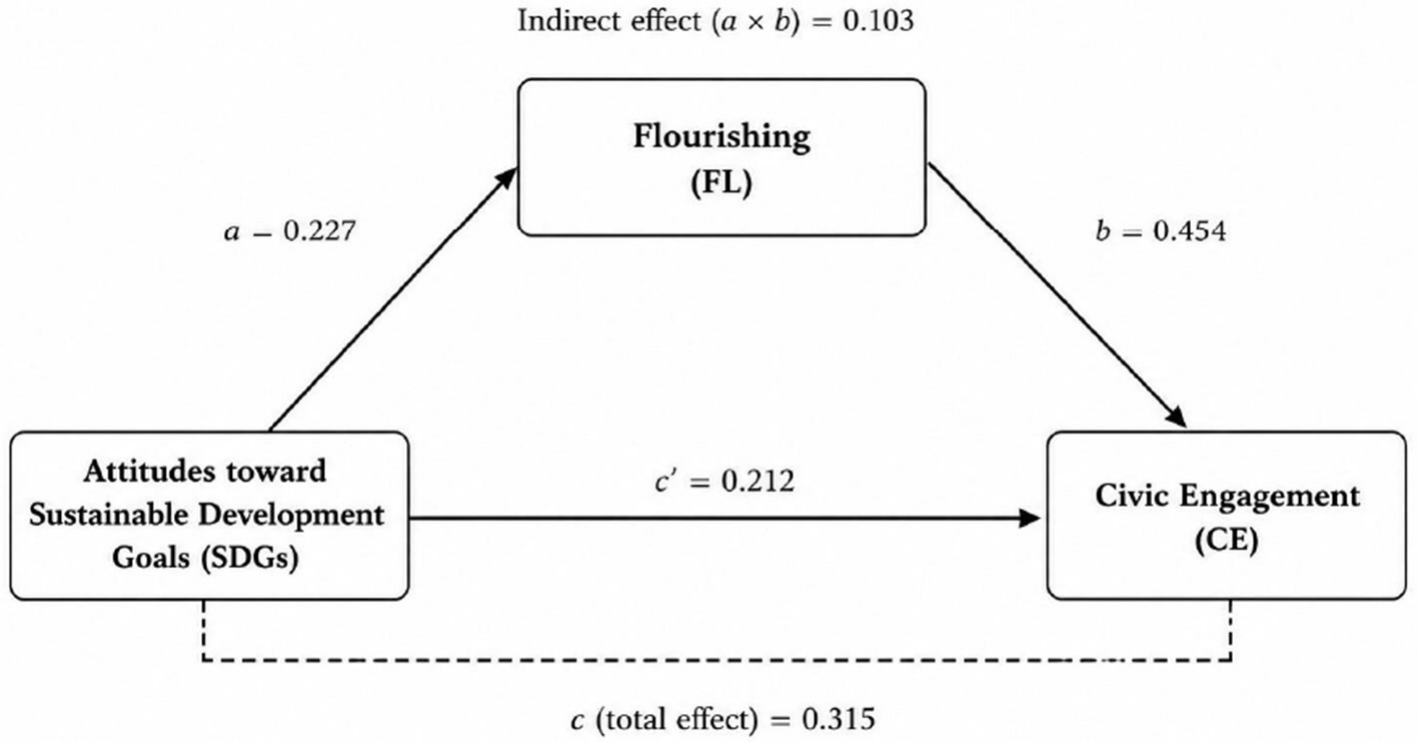
Final mediation model of the relationship between attitudes toward the Sustainable Development Goals (SDGs), flourishing, and civic engagement. Standardized coefficients (β) are reported. The indirect effect was estimated using bootstrapping with 5,000 resamples and 95% confidence intervals. a, effect of attitudes toward the SDGs on flourishing; b, effect of flourishing on civic engagement; c', direct effect of attitudes toward the SDGs on civic engagement controlling for flourishing; c, total effect.

The SDGs also demonstrated a positive predictive effect on flourishing (β = 0.227, *t* = 6.532, and *p* < 0.01), with a 95% CI of [0.124, 0.331]. Finally, FL showed a positive predictive effect on CE (β = 0.454, *t* = 8.804, and *p* < 0.01), with a 95% CI of [0.352, 0.555]. Furthermore, the 95% bootstrapped confidence intervals for the direct effect of the SDGs on CE and the indirect (mediating) effect of FL did not include zero (Table 3). These findings suggest that attitudes toward the SDGs directly predict civic engagement through the mediating effect of flourishing. The direct effect was 0.212, and the indirect (mediating) effect was 0.103, accounting for 67.3% and 32.7% of the total effect (0.315), respectively. Figure 2 shows the final mediation model.

**Table 3:** Analysis of total effect, direct effect, and mediating effect.

Predictive effects	Effect	Boot SE	Boot LLCI	Boot ULCI	Percentage of effect value
Total effect	0.315	0.459	0.225	0.406	
Direct effect	0.212	0.411	0.131	0.293	67.3
Mediating effect of flourishing	0.103	0.034	0.052	0.162	32.7

## Discussion

The findings of the present study provide a robust empirical foundation for understanding the psychological mechanisms that relation with civic engagement (CE) among university students in the context of the United Nations' 2030 Agenda. By examining the relationship between attitudes toward Sustainable Development Goals (SDGs) and active participation, mediated by the construct of flourishing, this research illuminates a critical way for institutional interventions aimed at fostering a more sustainable society. The results demonstrate that while pro-SDG attitudes are a significant direct predictor of civic engagement (beta = 0.315, *p* < 0.01), their impact is substantially channeled through the individual's state of flourishing, which accounts for 32.7% of the total effect (Delli Carpini, 2000; Ehrlich, 2000).

### The predictive power of SDG attitudes

The direct association between positive attitudes toward the SDGs and civic engagement underscores the importance of evaluative judgment in the mobilization of youth behavior. In this study, attitudes represent not merely an abstract approval of global goals but a predisposed psychological tendency to evaluate sustainability-related issues with favor. The statistical significance of this relationship (beta = 0.212 after controlling for flourishing) suggests that the moral and social imperatives embedded within the 17 SDGs—ranging from poverty eradication to climate action—act as a cognitive ingredient for collective action.

This finding is particularly salient when compared to the Knowledge, Attitude, and Practice (KAP) studies conducted in other international contexts. For instance, research at the University of Malaya (Afroz and Ilham, 2020) identified a significant disconnect between students' knowledge of the SDGs and their actual practice, with a weak negative correlation (*r* = −0.264) observed between these two variables. However, the same study found a strong positive correlation (*r* = 0.440) between attitude and practice. Our results corroborate this global trend: while raw information about sustainability may not be sufficient to incentive engagement, the internalization of these goals as positive values is a critical behavioral determinant (Sagiv et al., 2022).

The Spanish university context, specifically within Andalusia, provides a unique environment for the observation of these dynamics. Emerging adults (aged 18–29) are in a transitional phase characterized by the consolidation of personal, political, and civic identities. During this period, university socialization environments serve as key incubators where students develop social competencies and interpersonal trust. Our data suggest that when students perceive the SDGs as relevant to their own future and the wellbeing of their communities, they are more likely to take action on public issues.

### From flourishing to engagement

A central contribution of this study is the identification of flourishing as a critical mediator. Flourishing, conceptualized as a state of elevated emotional, psychological, and social wellbeing, represents the “psychological capital” necessary for sustained civic involvement (Keyes, 2005; Seligman, 2011). The indirect effect found in our analysis (0.103) suggests that psychological flourishing may help explain the association between positive SDG attitudes and civic engagement among university students. Rather than implying a direct causal sequence where flourishing “fuels” engagement, these findings indicate that optimal eudaimonic wellbeing acts as an important intermediate psychological resource that is concurrently present when students translate their pro-sustainability attitudes into active community participation.

This mediation can be explained through the six interrelated attributes of flourishing identified by Keyes (2005). When students align themselves with the SDGs, they may experience a heightened sense of social contribution (the feeling that one has something important to offer society) and social coherence (the perception that the social world is intelligible and meaningful). This psychological alignment reduces the sense of alienation and powerlessness that often discourages young people from participating in civic life.

Furthermore, our findings align with research on Italian adolescents (Albanesi et al., 2007), which demonstrated that a sense of community and social wellbeing are strong predictors of prosocial behaviors. In the Spanish sample, flourishing acts as a eudaimonic resource; students who are “flourishing” possess richer social connections and experience fewer limitations in their daily activities, making them more resilient to the stresses associated with activism and community service. This is crucial given the reported 71% increase in serious psychological distress among young adults over the last decade, often attributed to the pressures of digital environments and global instability (Twenge et al., 2019).

Our findings should also be interpreted through the lens of Value-Belief-Norm (VBN) theory. As suggested by research on the Chinese public, individuals with altruistic and biospheric value orientations are significantly more supportive of SDGs (Guan and Zhang, 2023). These values emphasize the welfare of fellow humans and the non-human world, which are foundational to the 2030 Agenda.

In the Spanish sample, we can infer that pro-SDG attitudes are rooted in these altruistic values. When these values are internalized, they form “personal norms”—internalized feelings of moral obligation that compel the individual to act. The role of flourishing here is transformative: it takes the “obligation” of a norm and turns it into a source of psychological fulfillment. By engaging in sustainability-oriented actions, students are not just fulfilling a duty; they are realizing their potential and enhancing their subjective wellbeing.

### Institutional implications for higher education

The significant mediating role of flourishing suggests that universities should prioritize student wellbeing not just as a health outcome, but as a supportive eudaimonic pathway that may facilitate student community involvement and active citizenship. Campuses that foster a sense of belonging and provide opportunities for meaningful social contribution are indirectly preparing their students for global citizenship (Wakkee et al., 2019).

Case studies from Brazil illustrate how universities can act as civic agents by localizing the SDGs and training key actors in the community (Minillo and Menezes, 2021). Similarly, incorporating “testimonio”—narratives of lived experience with social issues—can be a powerful pedagogical tool to develop the reflexivity and empathy required for transnational activism (Ortbals et al., 2021). Our data suggest that Spanish universities could enhance sustainability-oriented participation by creating “habitats for experimental enactment,” where students can practice civic skills in a safe, supportive environment that promotes flourishing.

### Limitations

While the use of SPSS PROCESS Macro (Model 4) and bootstrapping provides high statistical confidence in the mediation findings, several limitations must be showed. The sample consists predominantly of women (164 out of 215), which may influence the results. Research has suggested that gender can moderate the relationship between engagement and wellbeing, with some evidence indicating that men may reap different mental health benefits from club membership or activism than women (Brown et al., 2017; Landstedt et al., 2016).

Additionally, the cross-sectional and self-reported nature of our data represents a key limitation, as it prevents us from establishing temporal precedence or making definitive causal inferences. It is highly plausible that the relationship operates within a bidirectional eudaimonic loop—an upward spiral where active civic participation in turn enhances and reinforces students' flourishing over time.

Consequently, our results must be interpreted cautiously as positive associations, and future longitudinal or experimental studies are crucially required to rigorously test these underlying causal mechanisms over the course of a student's academic life and transition into adulthood (Piliavin and Siegl, 2007).

## Conclusions

This study identifies a critical psychological pathway toward achieving the Sustainable Development Goals through youth mobilization. The empirical evidence demonstrates that positive attitudes toward the SDGs are vital predictors of civic engagement, and that this relationship is significantly strengthened by the presence of psychological flourishing. Flourishing accounts for approximately one-third of the total effect, highlighting that a student's internal sense of social and emotional wellbeing is positively associated with and may play a crucial role in facilitating active, sustainable participation, rather than acting as a strict prerequisite (Keyes, 2005).

It is important to highlight that the finding regarding the mediation of flourishing (32.7%) represents a significant advance compared to other previous models. For example, Fenn et al. (2023) observed that in young people without university education, the relationship with wellbeing is primarily mediated by civic efficacy and sense of purpose. Our study demonstrates that in the university context, holistic flourishing is a more comprehensive vehicle for activating social engagement.

Furthermore, while research such as that by Albanesi et al. (2007) emphasizes that “sense of community” is the strongest predictor of social wellbeing in adolescents; our results indicate that positive attitudes toward the SDGs act as a cognitive driver for generating that wellbeing in emerging adulthood. This is key to closing the gap between theoretical knowledge and sustainable practice identified by Afroz and Ilham (2020) in other academic contexts. By promoting flourishing, universities can bridge the gap between abstract sustainability values and localized civic practice.

To create an engaged citizenry, it is insufficient to provide only knowledge about global goals. Instead, institutions must cultivate the psychological resources—social contribution, integration, and efficacy—that allow students to see themselves as meaningful agents of change (Wakkee et al., 2019). Positive pedagogy frameworks, such as service-learning and community mentoring, offer ideal contexts for students to acquire academic competencies, cultivate personal flourishing, and provide high-value contributions to their social environments.

Furthermore, the research supports the integration of wellbeing-centered interventions within the university curriculum (Zhao et al., 2025). Specifically, we now highlight the integration of positive psychology interventions (PPIs), strengths-based positive education, and high-impact experiential pedagogies such as service-learning (SL) and community youth mentoring programs. These structured academic spaces provide the ideal habitat for students to develop their personal flourishing (PERMA pillars) while actively applying sustainability concepts to local community needs. As the world faces increasing climate and social challenges, the eudaimonic health of the next generation will be the most critical determinant of a resilient and participatory society.

## Data Availability

The original contributions presented in the study are included in the article/supplementary material, further inquiries can be directed to the corresponding author.

## References

[B1] Adler R. P. Goggin J. (2005). What do we mean by “civic engagement”? J. Transform. Educ. 3, 236–253. doi: 10.1177/1541344605276792

[B2] Afroz N. Ilham Z. (2020). Assessment of knowledge, attitude and practice of University students towards Sustainable Development Goals (SDGs). J. Indones. Sustain. Dev. Plan. 1, 31–44. doi: 10.46456/jisdep.v1i1.12

[B3] Aguirre-Urreta M. I. Hu J. (2019). Detecting common method bias: performance of the harman's single-factor test. ACM SIGMIS Database Adv. Inf. Syst. 50, 45–70. doi: 10.1145/3330472.3330477

[B4] Albanesi C. Cicognani E. Zani B. (2007). Sense of community, civic engagement and social well-being in Italian adolescents. J. Community Appl. Soc. Psychol. 17, 387–406 doi: 10.1002/casp.903

[B5] Albright D. L. McDaniel J. T. Godfrey K. Thomas K. H. Fletcher K. L. Rosen G. (2020). Civic engagement among student veterans. J. Am. Coll. Health 68, 387–394. doi: 10.1080/07448481.2018.1559170 30681934

[B6] Alcántara-Rubio L. Valderrama-Hernández R. Solís-Espallargas C. Ruiz-Morales J. (2022). The implementation of the SDGs in universities: a systematic review. Environ. Educ. Res. 28, 1585–1615. doi: 10.1080/13504622.2022.2063798

[B7] Arnett J. J. (2007). Emerging adulthood: what is it, and what is it good for? Child Dev. Perspect. 1, 68–73. doi: 10.1111/j.1750-8606.2007.00016.x

[B8] Ballard P. J. Hoyt L. T. Pachucki M. C. (2019). Impacts of Adolescent and young adult civic engagement on health and socioeconomic status in adulthood. Child Dev. 90, 1138–1154. doi: 10.1111/cdev.12998 29359473

[B9] Bobek D. Zaff J. Li Y. Lerner R. M. (2009). Cognitive, emotional, and behavioral components of civic action: towards an integrated measure of civic engagement. J. Appl. Dev. Psychol. 30, 615–627. doi: 10.1016/j.appdev.2009.07.005

[B10] Brown N. J. L. Lomas T. Eiroa-Orosa F. J. (2017). The Routledge International Handbook of Critical Positive Psychology. London: Routledge. doi: 10.4324/9781315659794

[B11] Carlsen L. Bruggemann R. (2022). The 17 United Nations' sustainable development goals: a status by 2020. Int. J. Sustain. Dev. World Ecol. 29, 219–229. doi: 10.1080/13504509.2021.1948456

[B12] Catalino L. I. Fredrickson B. L. (2011). A Tuesday in the life of a flourisher: the role of positive emotional reactivity in optimal mental health. Emotion 11, 938–950. doi: 10.1037/a0024889 21859208 PMC3160725

[B13] Chan W. Y. Ou S. R. Reynolds A. J. (2014). Adolescent civic engagement and adult outcomes: an examination among urban racial minorities. J. Youth Adolesc. 43, 1829–1843. doi: 10.1007/s10964-014-0136-5 24878896 PMC4192036

[B14] Datu J. A. (2018). Flourishing is associated with higher academic achievement and engagement in Filipino undergraduate and high school students. J. Happiness Stud. 19, 27–39. doi: 10.1007/s10902-016-9805-2

[B15] Delli Carpini M. X. (2000). Gen.com: youth, civic engagement, and the new information environment. Polit. Commun. 17, 341–349. doi: 10.1080/10584600050178942

[B16] Diener E. Wirtz D. Biswas-Diener R. Tov W. Kim-Prieto C. Choi D.-w. . (2009). “New measures of well-being,” in Assessing Well-Being: The Collected Works of Ed Diener, ed. E. Diener (Dordrecht: Springer Science + Business Media), 247–266. doi: 10.1007/978-90-481-2354-4_12

[B17] Ding N. Berry H. L. O'Brien L. V. (2015). One-year reciprocal relationship between community participation and mental wellbeing in Australia: a panel analysis. Soc. Sci. Med. 128, 246–254. doi: 10.1016/j.socscimed.2015.01.022 25633762

[B18] Dolan P. (2022). Social support, empathy, social capital and civic engagement: intersecting theories for youth development. Educ. Citizsh. Soc. Jus. 17, 255–267. doi: 10.1177/17461979221136368

[B19] Ehrlich T. (2000). Civic Responsibility and Higher Education. Phoenix, AZ: Oryx. doi: 10.5771/9781461636625

[B20] Fenn N. Robbins M. L. Harlow L. Pearson-Merkowitz S. (2021). Civic engagement and well-being: examining a mediational model across gender. Am. J. Health Promot. 35, 917–928. doi: 10.1177/08901171211001242 33739159

[B21] Fenn N. Sacco A. Monahan K. Robbins M. Pearson-Merkowitz S. (2024). Examining the relationship between civic engagement and mental health in young adults: a systematic review of the literature. J. Youth Stud. 27, 558–587. doi: 10.1080/13676261.2022.2156779 38706784 PMC11068018

[B22] Fenn N. Yang M. Pearson-Merkowitz S. Robbins M. (2023). Civic engagement and well-being among non college young adults: investigating a mediation model. J. Community Psychol. 51, 2667–2685. doi: 10.1002/jcop.23033 36943410 PMC10629573

[B23] Fong C.-P. To S.-M. (2022). Civic engagement, social support, and sense of meaningfulness in life of adolescents living in Hong Kong: implications for social work practice. Child Adolesc. Soc. Work J. 41, 1–13. doi: 10.1007/s10560-022-00819-7 35095181 PMC8781701

[B24] Fredrickson B. L. (2004). The broaden-and-build theory of positive emotions. Philos. Trans. R. Soc. Lond. B Biol. Sci. 359, 1367–1378. doi: 10.1098/rstb.2004.1512 15347528 PMC1693418

[B25] Galvão T. G. Menezes H. Z. de Moraes L. E. Cabral R. Maurer R. L. Pereira R. S. . (2024). “Challenges and opportunities of a Brazilian network of universities for the SDG implementation,” in Sustainable Development Goals Series, eds. T. G. Galvao and H. Z. de Menezes (Cham: Springer International Publishing), 59–69. doi: 10.1007/978-3-031-59279-9_5

[B26] Guan T. Zhang Q. (2023). Value orientations, personal norms, and public attitude toward SDGs. Int. J. Environ. Res. Public Health 20:4031. doi: 10.3390/ijerph20054031 36901041 PMC10002065

[B27] Hansen J. A. Lehmann M. (2006). Agents of change: universities as development hubs. J. Clean. Prod. 14, 820–829. doi: 10.1016/j.jclepro.2005.11.048

[B28] Hayes A. F. (2012). PROCESS: A Versatile Computational Tool for Observed Variable Mediation, Moderation, and Conditional Process Modeling [White Paper]. Availble online at: http://www.afhayes.com/public/process2012.pdf (Accessed February 6, 2026).

[B29] Hone L. C. Jarden A. Schofield G. M. Duncan S. (2014). Measuring flourishing: the impact of operational definitions on the prevalence of high levels of wellbeing. Int. J. Wellbeing 4, 62–90.doi: 10.5502/ijw.v4i1.4

[B30] Hong X. Calderon A. Coates H. (2023). Universities and SDGs: evidence of engagement and contributions, and pathways for development. Policy Rev. High. Educ. 7, 56–77. doi: 10.1080/23322969.2022.2121311

[B31] Huppert F. A. So T. T. C. (2013). Flourishing across Europe: application of a new conceptual framework for defining well-being. Soc. Indic. Res. 110, 837–861. doi: 10.1007/s11205-011-9966-7 23329863 PMC3545194

[B32] Kaliyaperumal K. (2004). Guideline for conducting a knowledge, attitude and practice (KAP) study. AECS Illum. 4, 7–9.

[B33] Keyes C. L. M. (2002). The mental health continuum: from languishing to flourishing in life. J. Health Soc. Behav. 43, 207–222. doi: 10.2307/3090197 12096700

[B34] Keyes C. L. M. (2005). Mental illness and/or mental health? Investigating axioms of the complete state model of health. J. Consult. Clin. Psychol. 73, 539–548. doi: 10.1037/0022-006X.73.3.539 15982151

[B35] Keyes C. L. M. Dhingra S. S. Simoes E. J. (2010). Change in level of positive mental health as a predictor of future risk of mental illness. Am. J. Public. Health 100, 2366–2371. doi: 10.2105/AJPH.2010.192245 20966364 PMC2978199

[B36] Landstedt E. Almquist Y. B. Eriksson M. Hammarström A. (2016). Disentangling the directions of associations between structural social capital and mental health: longitudinal analyses of gender, civic engagement and depressive symptoms. Soc. Sci. Med. 163, 135–143 doi: 10.1016/j.socscimed.2016.07.005 27423294

[B37] Lerner R. M. Wang J. Champine R. B. Warren D. J. A. Erickson K. (2014). Development of civic engagement: theoretical and methodological issues. Int. J. Dev. Sci. 8, 69–79. doi: 10.3233/DEV-14130

[B38] Liu D. Wang Z. Wang Y. Yu W. Wang H. (2025). The relationship between social support and ostracism among college students: the mediating role of social connectedness and the moderating role of flourishing. Behav. Sci. 15:1198. doi: 10.3390/bs15091198 41009228 PMC12466604

[B39] Minillo X. K. Menezes H. (2021). “The university as a civic agent: promoting civic engagement and the UN SDGs in Northeastern Brazil” in Teaching Civic Engagement Globally, eds. E. Matto, A. R. M. McCartney, E. A. Bennion, and D. Simpson (Washington, DC: American Political Science Association), 37–52.

[B40] Morawska-Jancelewicz J. (2022). The role of universities in social innovation within quadruple/quintuple helix model: practical implications from Polish experience. J. Knowl. Econ. 13, 2230–2271. doi: 10.1007/s13132-021-00804-y 40477557 PMC8214917

[B41] Nelson L. J. Padilla-Walker L. M. (2013). Flourishing and floundering in emerging adult college students. Emerg. Adulthood 1, 67–78. doi: 10.1177/2167696812470938

[B42] Ng T. Y. Ng T. K. Siu O. L. (2025). Enhancing mental well-being in university students through multicomponent low intensity positive education and the mediating role of civic engagement. Sci. Rep. 15:20871. doi: 10.1038/s41598-025-05960-8 40595984 PMC12217956

[B43] Nuangchalerm P. (2014). Self-efficacy and civic engagement in undergraduate students: empirical results of a service learning program in Thailand. Int. J. Serv. Learn. Eng. Humanit. Eng. Soc. Entrep. 9, 150–156. doi: 10.24908/ijsle.v9i2.5456

[B44] O'Dwyer M. Filieri R. O'Malley L. (2023). Establishing successful university–industry collaborations: barriers and enablers deconstructed. J. Technol. Transf. 48, 900–931. doi: 10.1007/s10961-022-09932-2

[B45] Ortbals C. D. Poloni-Staudinger L. M. Jenkins S. Rincker M. (2021). “Stop training global political hobbyists! Teaching students how to engage in transnational feminism,” in Teaching Civic Engagement Globally, eds. E. C. Matto, A. R. M. McCartney, E. A. Bennion, and D. Simpson (Washington, DC: American Political Science Association), 21–43.

[B46] Piliavin J. A. Siegl E. (2007). Health benefits of volunteering in the Wisconsin Longitudinal Study. J. Health Soc. Behav. 48, 450–464. doi: 10.1177/002214650704800408 18198690

[B47] Pozo C. Bretones B. (2019). Spanish Version of the Flourishing Scale (FS) on the parents of children with cancer: a validation through Rasch analysis. Front. Psychol. 10:35. doi: 10.3389/fpsyg.2019.00035 30740074 PMC6355706

[B48] Pozo C. Bretones B. Vázquez M. Á. (2021). When your child has cancer: a path-analysis model to show the relationships between flourishing and health in parents of children with cancer. Int. J. Environ. Res. Public Health 18:12587. doi: 10.3390/ijerph182312587 34886313 PMC8657391

[B49] Pozo C. Garzón A. Bretones B. Ligia C. (2016). Propiedades psicométricas y dimensionalidad de “The Flourishing Scale” en población hispanohablante. Electron. J. Res. Educ. Psychol. 14, 175–182. doi: 10.14204/ejrep.38.15044

[B50] Sagiv I. B. Goldner L. Carmel Y. (2022). The civic engagement community participation thriving model: a multi-faceted thriving model to promote socially excluded young adult women. Front. Psychol. 13:955777. doi: 10.3389/fpsyg.2022.955777 36186320 PMC9521641

[B51] Seligman M. E. P. (2011). Flourish: A Visionary New Understanding of Happiness and Well-Being. New York, NY: Free Press.

[B52] Spearman C. (2010). The proof and measurement of association between two things. Int. J. Epidemiol. 39, 1137–1150. doi: 10.1093/ije/dyq191 21051364

[B53] Tang X. Duan W. Wang Z. Liu T. (2016). Psychometric evaluation of the simplified Chinese version of flourishing scale. Res. Soc. Work. Pract. 26, 591–599. doi: 10.1177/1049731514557832

[B54] Twenge J. M. Cooper A. B. Joiner T. E. Duffy M. E. Binau S. G. (2019). Age, period, and cohort trends in mood disorder indicators and suicide-related outcomes in a nationally representative dataset, 2005–2017. J. Abnorm. Psychol. 128, 185–199. doi: 10.1037/abn0000410 30869927

[B55] United Nations General Assembly (2015). Transforming Our World: The 2030 Agenda for Sustainable Development (A/RES/70/1). Available online at: https://sdgs.un.org/2030agenda (Accessed February 26, 2026).

[B56] Wakkee I. van der Sijde P. Vaupell C. Ghuman K. (2019). The university's role in sustainable development: activating entrepreneurial scholars as agents of change. Technol. Forecast. Soc. Chang. 141, 195–205. doi: 10.1016/j.techfore.2018.10.013

[B57] WMA (2013). Declaration of Helsinki. Ethical Principles for Medical Research Involving Human Subjects, 64th Edn. Fortaleza: WMAGeneral Assembly. Available online at: https://jamanetwork.com/journals/jama/fullarticle/1760318/ (Accessed February 26, 2026).

[B58] Wodika A. B. Middleton W. K. (2020). Climate change advocacy: exploring links between student empowerment and civic engagement. Int. J. Sustain. High. Educ. 21, 1209–1231. doi: 10.1108/IJSHE-03-2020-0091

[B59] Zhao G. X. Zhao M. Y. Bean C. R. Robbins A. S. Mahrer N. E. (2025). Well-being interventions in U.S. colleges: a scoping review from a positive higher education perspective. Front. Psychol. 16:1601955. doi: 10.3389/fpsyg.2025.1601955 40949350 PMC12426774

